# Quantifying and exploring camouflaging in men and women with autism

**DOI:** 10.1177/1362361316671012

**Published:** 2016-11-29

**Authors:** Meng-Chuan Lai, Michael V Lombardo, Amber NV Ruigrok, Bhismadev Chakrabarti, Bonnie Auyeung, Peter Szatmari, Francesca Happé, Simon Baron-Cohen, MRC AIMS Consortium

**Affiliations:** 1 Centre for Addiction and Mental Health, Canada; 2 The Hospital for Sick Children, Canada; 3 University of Toronto, Canada; 4 University of Cambridge, UK; 5 National Taiwan University Hospital, Taiwan; 6 University of Cyprus, Cyprus; 7 University of Reading, UK; 8 The University of Edinburgh, UK; 9 King’s College London, UK; 10 Cambridgeshire and Peterborough NHS Foundation Trust, UK

**Keywords:** adults, autism, brain structure, camouflage, camouflaging, cognition, coping, gender, sex, sex differences

## Abstract

Autobiographical descriptions and clinician observations suggest that some individuals with autism, particularly females, ‘camouflage’ their social communication difficulties, which may require considerable cognitive effort and lead to increased stress, anxiety and depression. Using data from 60 age- and IQ-matched men and women with autism (without intellectual disability), we operationalized camouflaging in adults with autism for the first time as the quantitative discrepancy between the person’s ‘external’ behavioural presentation in social–interpersonal contexts (measured by the Autism Diagnostic Observation Schedule) and the person’s ‘internal’ status (dispositional traits measured by the Autism Spectrum Quotient and social cognitive capability measured by the ‘Reading the Mind in the Eyes’ Test). We found that the operationalized camouflaging measure was not significantly correlated with age or IQ. On average, women with autism had higher camouflaging scores than men with autism (Cohen’s *d* = 0.98), with substantial variability in both groups. Greater camouflaging was associated with more depressive symptoms in men and better signal-detection sensitivity in women with autism. The neuroanatomical association with camouflaging score was largely sex/gender-dependent and significant only in women: from reverse inference, the most correlated cognitive terms were about emotion and memory. The underlying constructs, measurement, mechanisms, consequences and heterogeneity of camouflaging in autism warrant further investigation.

## Introduction

Autism spectrum condition/disorder (henceforth ‘autism’) has a life-long impact on individual development. Adult presentation and outcome vary substantially (Howlin and Moss, 2012). Those who are diagnosed in childhood tend to show reduced autistic symptoms over time, but only a minority show satisfactory social functioning (Howlin et al., 2013). In contrast to individuals who receive diagnoses in childhood, some individuals are only identified later in life and may ‘fly under the radar’ for many years partly because of learnt strategies to conceal social difficulties. These late-diagnosed individuals tend to suffer from concurrent mental health challenges potentially related to long-term stress in adaptation to daily life in the society (Lai and Baron-Cohen, 2015). Given long-standing environmental support but also pressure to ‘fit in’ with neurotypical social communication, individuals with autism (irrespective of timing of diagnosis) may develop coping strategies over development. One such coping strategy is that they may ‘camouflage’ difficulties during social situations (Attwood, 2007) by either hiding behaviour that might be viewed as socially unacceptable or artificially ‘performing’ social behaviour deemed to be more neurotypical – they *Pretend to be Normal* (Willey, 1999).

Examples of camouflaging include making eye contact during conversation, using learned phrases or pre-prepared jokes in conversation, mimicking other’s social behaviour, imitating facial expressions or gestures, and learning and following social scripts (Lai and Baron-Cohen, 2015). One may also learn to consciously speak more quietly or not to stand too close to another person or not to make personal remarks, perhaps following feedback that these may be hurtful or uncomfortable for others or perhaps as a conscious goal to model their behaviour on a neurotypical peer in order to gain greater social acceptance. Autobiographical descriptions and clinician observations often suggest that camouflaging unfortunately comes at a cost: it often requires substantial cognitive effort, can be exhausting and may lead to increased stress responses, meltdown due to social overload, anxiety and depression, and even a negative impact on the development of one’s identity (Attwood, 2007; Boyd et al., 2011; Lai et al., 2011; Simone, 2010; Willey, 1999; Williams, 1992).

Camouflaging may also play a role in the observed male-preponderance in autism prevalence, if it is the case that females are more likely or more motivated to camouflage, and thereby go undetected and undiagnosed for longer. Thus, the male-preponderance may reflect aetiological sex/gender differences, but may also be a product of under- or misrecognition of autism in females, potentially associated with gender stereotypes and the historically male-based behavioural characterization of autism, with insufficient acknowledgement of how females may present some behaviours characteristic of autism in a qualitatively or quantitatively different way from their male counterparts – camouflaging being one example (Lai et al., 2015). Population-based data show that females are often diagnosed at later ages (Begeer et al., 2013; Giarelli et al., 2010; Rutherford et al., 2016; Shattuck et al., 2009) and less easily than males with autism (Russell et al., 2011; Wilson et al., 2016), unless there are concurrent behavioural or cognitive challenges (Dworzynski et al., 2012). One of the potential reasons for this may be the heightened tendency to camouflage difficulties in many females on the spectrum: when difficulties in social interaction and communication are masked, their signs of autism are less likely to be picked up by families, teachers or primary care providers in order to trigger an assessment. If the diagnostician further misses signs of camouflaging, superficially ‘typical’ non-verbal skills and social manner may be wrongly taken as evidence to rule out the presence of autism (Lai and Baron-Cohen, 2015).

In the 1980s, investigating sex/gender ratio in the autism spectrum, Wing (1981) wrote that ‘The possibility that girls with the triad of impairments who had higher levels of intelligence were missed in the search for cases has to be considered’ (p. 134). Ten years later, Gillberg (1991) noted that ‘Asperger syndrome can occur in girls […] on the surface, symptoms of impairment of social interaction might be less conspicuous than corresponding symptoms in boys’ (p. 129). He suggested that girls might have more advanced social skills to conceal their autistic characteristics. Attwood (2007) also pointed out in his highly influential book *The Complete Guide to Asperger’s Syndrome* that ‘Some girls and women with Asperger’s syndrome, and adults of considerable intellectual ability, can be more difficult to diagnose due to an ability to camouflage their difficulties’ (p. 40). Women with autism and their parents regularly echo this observation and consider camouflaging as one of the major reasons females on the autism spectrum often go under-recognized until they can no longer compensate (Ernsperger and Wendel, 2007; Hendrickx, 2015; Lawson, 1998; Miller, 2003; Simone, 2010; Willey, 1999; Zaks, 2006).

Clinicians and researchers have also increasingly described camouflaging in females, in contrast to males, with autism (Attwood, 2006; Gould and Ashton-Smith, 2011; Kopp and Gillberg, 1992; Lai et al., 2015; Marshall, 2015). Recent large-scale, population-based epidemiological studies (many of them included active case ascertainment) show a 2–3:1 male-preponderance compared to the widely cited 4–5:1 ratio from earlier studies (Baxter et al., 2015; Idring et al., 2012; Kim et al., 2011; Mattila et al., 2011; Zablotsky et al., 2015), possibly suggesting better recognition of females in recent years owing to increased clinical awareness or more sensitive measurement. Longitudinal population-based studies in the Nordic regions particularly confirm this trend (Jensen et al., 2014; Kocovska et al., 2012). Improving our understanding of camouflaging, along with other possible ‘female-phenotypes of autism’, may further facilitate the identification of masked symptoms and difficulties and enhance timely diagnosis and support.

Although camouflaging has been frequently described as a major characteristic of women with autism (e.g. by the findings from the European Union (EU)-funded *Autism in Pink* project, http://autisminpink.net/), it has received surprisingly limited systematic scientific investigation. In a recent qualitative study, Tierney et al. (2016) interviewed 10 teenage girls with autism on the social challenges associated with adolescence and analysed the data using Interpretative Phenomenological Analysis. These teenage girls reported that they developed explicit strategies to manage social relationships, in particular imitation and masking. Hiller et al. (2014) compared school-age boys and girls who were clinically diagnosed with autism and discovered that they met the clinical criteria in somewhat different ways. Notably, some differences may underpin or reflect higher camouflaging in females. For example, girls were more likely to be able to integrate non-verbal and verbal behaviours, to have better imagination (at least at face value), to maintain a reciprocal conversation and to initiate (but not maintain) friendships. These characteristics seem to have ecological impacts, as school teachers reported far fewer concerns about girls than boys with autism regarding their social skills, friendship and externalizing behavioural problems (Hiller et al., 2014).

Head and colleagues found that teenage girls with autism scored higher on the Friendship Questionnaire (indicative of better/more friendship) than did teenage boys with autism and at a level comparable to that of typically developing teenage boys. One interpretation is that females with autism may ‘develop a capacity to camouflage or hide their social insecurities in order to fit in’ (Head et al., 2014: 6). Lai et al. (2011) alluded to higher camouflaging in women than men with autism based on the observation that, given similar levels of childhood autistic symptoms measured by the Autism Diagnostic Interview–Revised (ADI-R), women with autism tended to show less pronounced autistic features in interpersonal–social contexts as measured by the Autism Diagnostic Observation Schedule (ADOS).

By analysing behaviour from the demonstration activities in the ADOS-2, Rynkiewicz et al. (2016) found girls with autism used gestures more vividly than boys with autism and suspected this to be one component of enhanced camouflaging in females which ‘may pose risk of underdiagnosis or not receiving the appropriate diagnosis for this population’ (p. 6). Finally, Lehnhardt and colleagues studied cognitive profiles in late-diagnosed men and women with autism, both presumably missing early diagnosis partly due to camouflaging. They found that men with autism had higher verbal abilities than women with autism, whereas women with autism had higher processing speed and better executive function than men with autism. They proposed that this sex/gender-differential finding might indicate ‘different strategies being applied to camouflage the autistic background during childhood and adolescence’ (Lehnhardt et al., 2016: 150).

These pioneering studies indicate that camouflaging can be conceptualized as using learned social communicative behaviours (e.g. imitation, gestures and conversation skills) to mask underlying difficulties related to autism. Females with autism tend to employ more camouflaging than males with autism. This skill is probably supported by verbal ability and executive function. Since camouflaging involves real-time monitoring of the environment to make careful and appropriate responses, we hypothesize that in order to successfully camouflage, at the cognitive level one requires sensitive monitoring of the environment (i.e. being highly vigilant) and/or a more conservative response strategy (i.e. being highly cautious). Nevertheless, camouflaging may also be supported by other skills deemed to be relevant by its definition, such as social imitation ability, and/or other as yet unrecognized cognitive or behavioural abilities.

None of the above studies have operationalized and quantified camouflaging in autism or clarified its neurocognitive correlates. We consider there are at least two complementary approaches needed to advance our understanding. The first is to take a *grounded theory* approach (Glaser and Strauss, 2009), starting from research questions such as ‘what is camouflaging in autism?’ and ‘what are the required abilities and skills for camouflaging?’ and to collect qualitative data to inform concept formation. The second is to take a positivist approach and operationalize camouflaging using existing, standardized measures and to test for relevant hypotheses derived from the observations and findings summarized above. Here we take the latter approach, using existing standardized and validated measures to (1) derive an estimate of camouflaging in adults with autism, (2) compare camouflaging between males and females, (3) test whether more camouflaging is associated with more severe anxiety and depression and (4) test whether more camouflaging is associated with better verbal ability, better signal detection from background events and more conservative responses. In addition, in a hypothesis-free manner, we explore the neuroanatomical correlates of individual differences in camouflaging and then use the ‘big data’ from the neuroscience literature to draw a ‘reverse inference’ to identify other potentially associated cognitive correlates of camouflaging.

## Methods

### Participants

Participants comprised 30 adult females and 30 adult males with autism (none with intellectual disability) matched for age (18–49 years), verbal IQ (VIQ), performance IQ (PIQ) and full-scale IQ (FIQ). All participants had a formal clinical diagnosis of International Classification of Diseases, Tenth Edition (ICD-10; World Health Organization (WHO), 1992) childhood autism or Asperger’s syndrome and/or Diagnostic and Statistical Manual of Mental Disorders (4th ed., text rev.; DSM-IV-TR; American Psychiatric Association (APA), 2000) autistic disorder or Asperger’s disorder, as assessed by a psychiatrist or clinical psychologist in the National Health Service, United Kingdom. Additionally, all but two participants reached the diagnostic algorithm cut-offs on the ADI-R (Lord et al., 1994). The two exceptions were female participants where ADI-R was unavailable due to childhood caregivers being unable to be interviewed. One of these individuals scored above the cut-off for ‘autism spectrum’ on the ADOS (Lord et al., 2000) and the other was positive for a diagnosis on the Adult Asperger Assessment (AAA) which incorporates caregiver reports of childhood behaviours and developmental history (Baron-Cohen et al., 2005). Scoring one point below in only one of the three core symptom domains of ADI-R was permitted, to allow for possible underestimation of early developmentally atypical behaviours in the recall of caregivers whose children are now adults. ADOS module 4 was performed, but the score was not used as an inclusion criterion.

Behavioural, cognitive and neuroanatomical characterizations of this cohort have been reported previously (Ecker et al., 2012, 2013; Lai et al., 2011, 2012, 2013; Wilson et al., 2014), along with detailed project and recruitment information. The sample included in this study comprises the autism groups reported in a previous neuroimaging study (Lai et al., 2013).

### Behavioural and cognitive measures

All participants were assessed using the Wechsler Abbreviated Scale of Intelligence (Wechsler, 1999) for estimation of VIQ, PIQ and FIQ. ADI-R was conducted to assess childhood autism characteristics (Lord et al., 1994). Module 4 of the ADOS (Lord et al., 2000) was used to quantify current, adult (‘external’) behavioural characteristics related to autism. The ADOS is a standardized activity- and interview-based semi-structured assessment for current behavioural presentation in a quasi-natural, interpersonal context. Behaviours of the participant were coded immediately after the assessment session into 31 items, of which 16 were entered into the ‘diagnostic algorithm’. The diagnostic algorithm score quantifies the adult’s cardinal social interactive and communicative behaviours associated with autism.

Among a battery of self-report questionnaires obtained in this project (Lai et al., 2011), the Autism Spectrum Quotient (AQ) (Baron-Cohen et al., 2001b) was selected to measure participants’ self-reflection (‘internal’ perception) of their personal characteristics related to autism. The AQ contains 50 items measuring autistic-like traits in terms of social skills, attention switching, attention to detail, communication and imagination. Among a battery of cognitive tests in this project, the ‘Reading the Mind in the Eyes’ Test (RMET) (Baron-Cohen et al., 2001a, 2015) was selected to measure participants’ actual (‘internal’) capability in advanced mentalizing and complex emotion recognition. The 36-item RMET requires participants to infer mental status solely from photos of a person’s eyes and immediate surrounding areas of the face.

Based on our hypotheses, to test for the clinical and cognitive correlates of camouflaging, we selected the 21-item Beck Anxiety Inventory (BAI; Beck et al., 1988) to measure anxiety symptoms and the 21-item Beck Depression Inventory (BDI; Beck et al., 1961) to measure symptoms of depression. VIQ was selected to indicate the verbal ability of an individual. From our available cognitive measures, performance on executive function tasks most closely reflects our construct of interest (i.e. monitoring the environment and patterns of behavioural response). We therefore selected an online version of the Go/No-Go task and derived performance measures using the signal-detection theory (SDT) framework (Green and Swets, 1966), namely, *sensitivity* (*d′* = *Z*_Hit_ − *Z*_FA_, where *Z*_Hit_ is the corresponding *Z* value in the normal distribution for the probability of *Hit*, that is, signal present and the response is ‘present’, and *Z*_FA_ is the same for *False Alarm*, that is, signal absent but the response is ‘present’) and *criterion* (*response bias*) (*C* = −0.5 × (*Z*_Hit_ + *Z*_FA_)). *Sensitivity d′* indicates the participant’s ability to discriminate signal from background noise, and *criterion C* quantifies how liberal (i.e. *C* < 0) or conservative (i.e. *C* > 0) the response strategy (bias) is. Additional details of the implementation of cognitive tasks were reported earlier (Lai et al., 2012).

### Operationalizing camouflaging using standardized measures

As camouflaging could be defined as (consciously or unconsciously) *compensating* for and/or *masking* difficulties in social and interpersonal situations, we operationalized camouflaging as *the discrepancy between the person’s ‘external’ behavioural presentation in social–interpersonal contexts and the person’s ‘internal’ status (i.e. dispositional traits and/or social cognitive capability)*.

We used the ADOS diagnostic algorithm score as reflecting external presentation and both the AQ score and RMET correct score as reflecting internal status (self-rated dispositional traits and performance-based socio-cognitive capability, respectively). We used the ADOS diagnostic algorithm score to characterize one’s behavioural presentation because it is the only psychometrically tested, reliable measure of social communication behaviours in interpersonal contexts for individuals with autism. Although we have previously questioned the validity of using ADOS module 4 diagnostic algorithm cut-off for making diagnostic judgement for autism in adults without intellectual disability, particularly in females, we do not question the validity of ADOS in quantitatively measuring and describing cardinal social communication behaviours (Lai et al., 2011). The internal (i.e. latent) status of autism ideally is captured by a wide array of measures on the cognitive and psychological characteristics of the individual. Relying on one particular measure risks biases resulting from the measure’s inherent limitation (e.g. a self-report measure is dependent on one’s perception of their own behavioural/cognitive styles). We were confined by available data but were able to capture two key aspects, namely, one’s perception of their personal characteristics associated with autism and one’s cognitive performance on a mentalizing and emotion recognition task; both are integral parts in the assessment and understanding of individual characteristics of autistic people.

The three scores were first standardized (termed as *S*_ADOS_, *S*_AQ_ and *S*_RMET_) by mean-centring (to the whole autism sample in this study, *N* = 60) and scaling (i.e. divided by the maximum possible score of each) to generate uniformly scaled measures that can be arithmetically manipulated (i.e. added to or subtracted from each other); the uniformly scaled measures were derived in this way rather than using *z*-scores because *z*-standardization was problematic for the ADOS score, as (1) there was no available autistic population mean and standard deviation, and (2) sample mean and standard deviation were not valid substitutes here as the distribution was skewed. A first measure of camouflaging was quantified as the difference between self-rated autistic-like traits and external behaviours (CF1 = *S*_AQ_ − *S*_ADOS_), and a second measure between mentalizing ability and external behaviours (CF2 = −*S*_RMET_ − *S*_ADOS_); higher scores on CF1 and CF2 indicate more camouflaging. Finally, using principal component analysis, the first principal component score of CF1 and CF2 was taken as a single, parsimonious measure (the ‘camouflaging score’, CF) that incorporates information from all relevant measures for further analyses.

### Neuroimaging measures

Participants were scanned using a contemporary 3 T MRI scanner (GE Medical Systems HDx) fitted with an 8-channel receive-only RT head-coil using Driven Equilibrium Single Pulse Observation of T_1_ (DESPOT1) (Deoni et al., 2008). Simulated T_1_-weighted inversion recovery (IR) images were created via ImageJ. Pre-processing was conducted using the SPM12 software (Wellcome Trust Centre for Neuroimaging): tissue segmentation was done by Segment (previously New Segment), and the segmented grey matter (GM) images of the 60 participants were non-linearly normalized (with modulation) to the standard Montreal Neurological Institute (MNI) space using DARTEL (Ashburner, 2007) and smoothed with a 4-mm full-width at half maximum Gaussian kernel. Additional information about image acquisition has been reported previously (Lai et al., 2013).

### Statistical analysis

Statistical distribution of CF was examined for skewness and kurtosis. Independent-samples *t*-tests were used for comparisons between men and women with autism. Pearson’s correlation was used to examine the correlation patterns between CF and clinical/cognitive measures. Sex/gender difference in correlation patterns was tested by constructing a multiple regression model with clinical/cognitive measures as the dependent variable and CF, sex/gender and CF-by-sex/gender interaction as predictors, and then examining the significance of β on the interaction term: a significant interaction suggests that the correlation between CF and the dependent variable is dependent on sex/gender. Critical level for statistical significance was set at α = 0.05. These analyses were implemented using the IBM SPSS Statistics version 22.

Hypothesis-free exploration of the neuroanatomical correlates of CF was performed using mass-univariate tests with SPM12. Prior to statistical modelling, each modulated GM map was rescaled by individual total GM volume (i.e. voxel value divided by individual total GM volume) to derive relative regional GM volume estimates. First, for all participants, we fit a parsimonious general linear model (GLM) at each voxel, with sex/gender and CF as fixed factors, along with their interaction term, and age as a nuisance covariate. When significant CF-by-sex/gender interaction was found, we subsequently performed sex/gender-stratified whole-GM mass-univariate tests again to identify CF–GM volume association separately for the male and female groups; here, CF was the fixed factor and age was a nuisance covariate. All whole-GM voxel-level tests were restricted to voxels with a partial volume estimate >0.25. Multiple comparison correction was performed at the cluster level by controlling topological false discovery rate (FDR) calculated under Gaussian Random Field Theory (Chumbley and Friston, 2009), using a cluster-forming voxel-level height threshold of *p* < 0.01 for each contrast and a spatial extent threshold (corrected for non-stationarity (Hayasaka et al., 2004)) that ensures a cluster-wise FDR at *q* < 0.05.

## Results

### Statistical characteristics and sex/gender differences in the CF measure

**Figure 1.**
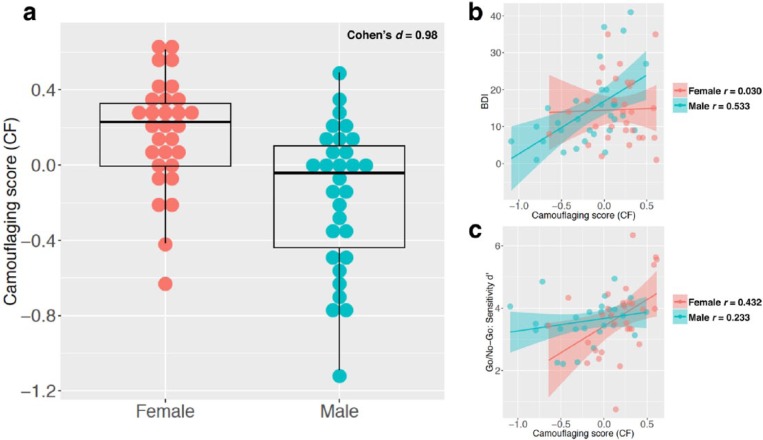
Sex/gender differences in camouflaging and its association with depressive symptoms and signal-detection sensitivity: (a) a dot and box-and-whisker plot showing the distribution of camouflaging (quantified by the measure CF) in men and women with autism; (b) CF-BDI score correlations stratified by sex/gender; (c) CF-sensitivity correlations stratified by sex/gender.

Sample characteristics are given in Table 1. In the present sample, CF did not significantly deviate from the normal distribution in either males with autism (skewness *z*-score = −1.351, kurtosis *z*-score = −0.363, Shapiro–Wilk test *p* = 0.288) or females with autism (skewness *z*-score = −1.710, kurtosis *z*-score = 1.013, Shapiro–Wilk test *p* = 0.213). Across the whole sample, CF was not significantly correlated with age (Pearson’s *r* = 0.188, *p* = 0.151), VIQ (*r* = 0.180, *p* = 0.168), PIQ (*r* = 0.053, *p* = 0.685), or FIQ (*r* = 0.136, *p* = 0.301); this was also the case when data were split into males only (age *r* = 0.303, *p* = 0.103; VIQ *r* = 0.095, *p* = 0.617; PIQ *r* = 0.099, *p* = 0.604; FIQ *r* = 0.120, *p* = 0.527) or females only (age *r* = 0.053, *p* = 0.783; VIQ *r* = 0.277, *p* = 0.138; PIQ *r* = 0.116, *p* = 0.540; FIQ *r* = 0.215, *p* = 0.253). Women with autism on average scored significantly higher on CF than men with autism (*p* < 0.001), with an effect size showing almost 1 standard deviation of difference (Cohen’s *d* = 0.98). However substantial variability on this measure was present in both males and females (see Figure 1(a)).

**Table 1.** Characteristics of the sample: men and women with autism.

Mean (SD) [range]^a^	Men (M) (*n* = 30)	Women (F) (*n* = 30)	Statistics^b^	Effect size (*d*)
Age (years)	27.2 (7.3)	27.8 (7.6)	ns (*p* = 0.761)	0.08
Verbal IQ	114.3 (12.9)	115.8 (13.1)	ns (*p* = 0.656)	0.12
Performance IQ	113.3 (15.0)	110.4 (16.7)	ns (*p* = 0.492)	0.18
Full-scale IQ	115.4 (14.1)	114.9 (13.8)	ns (*p* = 0.904)	0.04
ADI-R^c^				
Reciprocal social	18.0 (5.1) [10–27]	16.4 (4.3) [11–26]	ns (*p* = 0.215)	0.34
Communication	15.3 (3.5) [8–22]	13.1 (3.9) [8–22]	M > F (*p* = 0.029)	0.59
RRSB	5.6 (2.5) [2–10]	4.3 (1.7) [2–8]	M > F (*p* = 0.023)	0.60
ADOS				
Social communication	8.5 (5.0) [1–17]	4.3 (3.6) [0–13]	M > F (*p* < 0.001)	1.04
RRSB	1.0 (1.0) [0–4]	0.1 (0.3) [0–1]	M > F (*p* < 0.001)	1.25
Autism Spectrum Quotient	32.7 (7.3)	37.5 (6.7)	F > M (*p* = 0.010)	0.69
RMET correct score	22.8 (5.8)	23.4 (6.2)	ns (*p* = 0.700)	0.10
CF	−0.168 (0.388)	0.168 (0.294)	F > M (*p* < 0.001)	0.98
Go/No-Go task^d^				
Sensitivity, *d′*	3.60 (0.67)	3.71 (1.16)	ns (*p* = 0.660)	0.12
Response bias, *C*	−0.027 (0.198)	0.029 (0.232)	ns (*p* = 0.327)	0.26
Beck Depression Inventory	14.5 (10.3)	14.6 (9.0)	ns (*p* = 0.958)	0.01
Beck Anxiety Inventory	14.1 (9.9)	15.5 (10.1)	ns (*p* = 0.580)	0.14

SD: standard deviation; ns: non-significant (*p* > 0.05, two-tailed, not corrected for multiple comparisons); ADI-R: Autism Diagnostic Interview–Revised; RRSB: repetitive, restrictive and stereotyped behaviour; ADOS: Autism Diagnostic Observation Schedule.

a For ADI-R and ADOS scores.

b Independent-samples *t*-tests, except Mann–Whitney tests for ADOS scores (distribution significantly deviant from normal).

c *n* = 30 for men, *n* = 28 for women (*n* = 2 data missing due to childhood caregiver unavailability).

d *n* = 29 for men (*n* = 1 data missing due to technical failure), *n* = 30 for women.

### Testing correlations between CF and anxiety/depression

Across the whole sample, CF was positively correlated with the total score on the BDI (*r* = 0.301, *p* = 0.019) but not the BAI (*r* = 0.202, *p* = 0.121). When sex/gender-stratified, the CF-BDI correlation was significant in males (*r* = 0.533, *p* = 0.002) but not females (*r* = 0.030, *p* = 0.876). Male–female difference in correlation patterns approached significance (*p* = 0.068), testing the significance of CF-by-sex/gender interaction in a multiple regression model (see Figure 1(b)). Partialling out the effect of either PIQ or FIQ did not change any of these correlation patterns and significance.

### Testing correlations between CF and VIQ, signal detection and response bias

As reported above, across the whole sample, CF was not significantly correlated with VIQ (*r* = 0.180, *p* = 0.168); this was also true when examining only males (*r* = 0.095, *p* = 0.617) or only females (*r* = 0.277, *p* = 0.138). The Go/No-Go data for one male were missing due to technical failure, so data from only 29 men were included in the analysis. Across the whole sample, CF was positively correlated with Go/No-Go sensitivity *d′* (*r* = 0.311, *p* = 0.017) but not response bias *C* (*r* = 0.108, *p* = 0.416). When sex/gender-stratified, the CF-*d′* correlation was significant in females (*r* = 0.432, *p* = 0.017) but not males (*r* = 0.233, *p* = 0.223). Male–female difference in correlation patterns approached significance (*p* = 0.072) (see Figure 1(c)). Partialling out the effect of either PIQ or VIQ did not change any of these correlation patterns and significance.

### Exploring the neuroanatomical correlates of CF and using reverse inference to identify associated cognitive terms

**Figure 2.**
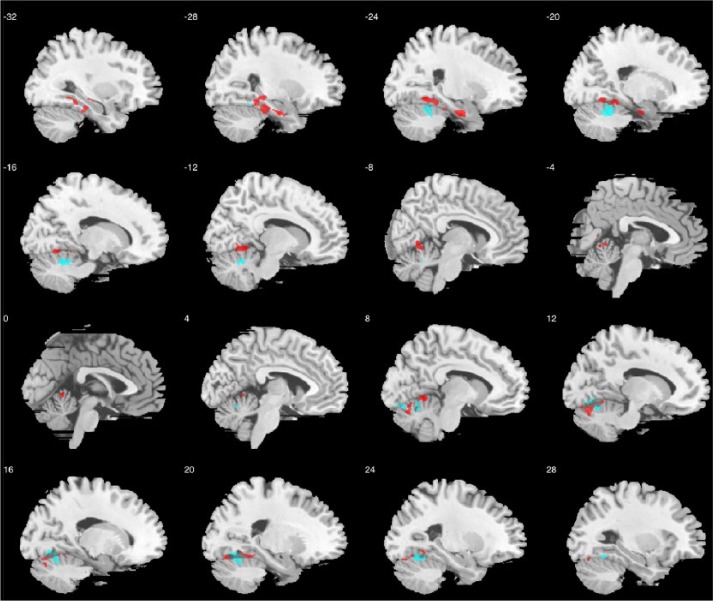
Sagittal slices illustrating grey matter regions showing sex/gender-differential associations between CF and regional volume (in red, involving left medial temporal lobe and cerebellum), overlaid with regions showing negative correlations between CF and regional volume in women with autism (in blue, involving cerebellum, occipital and medial temporal structures); threshold for visualization follows that described in the ‘Methods’ section.

The hypothesis-free, whole-GM voxel-based GLM identified no regions showing significant main effects of CF, but there were two significant clusters showing significant CF-by-sex/gender interaction, indicating sex/gender-dependent correlation patterns between CF and regional GM volume, at left medial temporal lobe (cluster size *k*_e_ = 4248, cluster-level FDR-corrected *q* < 0.001, peak-coordinate MNI (−26, −11, −28), *Z* = 4.04) and cerebellum (*k*_e_ = 3638, *q* < 0.001, peak-coordinate MNI (20, −70, −14), *Z* = 3.96), where increased CF was associated with decreased volume in females yet to a significantly different extent in males. To further identify anatomical correlates specific for females and males, we ran whole-GM voxel-based GLM again but separately by sex/gender. In females, there were significant negative correlations between CF and GM volume at bilateral cerebellum, occipital and medial temporal structures which substantially overlapped with the above-mentioned regions showing CF-by-sex/gender interaction (right-lateralized cluster *k*_e_ = 3605, *q* = 0.001, peak-coordinate MNI (15, −65, −8), *Z* = 4.04; left-lateralized cluster *k*_e_ = 1922, *q* = 0.027, peak-coordinate MNI (−15, −53, −25), *Z* = 3.81); no regions showed significant positive correlation with CF (see Figure 2). In males, there were no regions positively or negatively correlated with CF that survived multiple comparison corrections.

**Figure 3.**
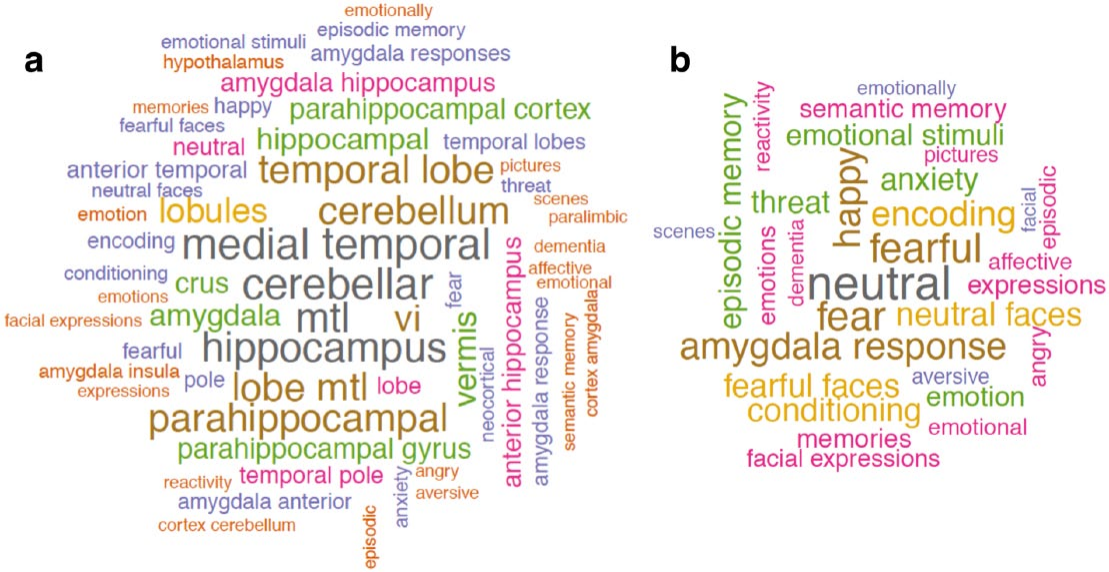
Word-clouds showing (a) the top 60 terms correlated with brain regions associated with camouflaging in women with autism, based on reverse inference using the Neurosynth Image Decoder, and (b) the top 30 terms after excluding anatomical terms.

In order to identify the terms in the scientific literature that are mostly associated with the identified voxels showing significant association with camouflaging in women with autism (i.e. making reasonable ‘reverse inference’ (Poldrack, 2006)), we submitted the statistical map to the Neurosynth Image Decoder (http://neurosynth.org/decode/; Gorgolewski et al., 2016) and visualized the top 60 terms showing highest correlation (*r* = 0.07–0.17) by a word-cloud (produced using R and the ‘wordcloud’ library; see Figure 3(a)). This qualitatively shows terms of anatomical regions (e.g. cerebellum, medial temporal lobe, para/hippocampus, amygdala) and terms about emotion and memory – see Figure 3(b) for the top 30 terms (*r* = 0.07–0.10) after filtering out anatomical terms.

## Discussion

In this exploratory study, we used an existing, well-characterized dataset in which standardized and widely used measures of behavioural characteristics, self-rated traits and ability to infer others’ mental states were available. We attempted to operationalize and quantify camouflaging in adults with autism, for the first time in the scientific literature, as the quantitative discrepancy between the person’s ‘external’ behavioural presentation in social–interpersonal contexts (measured by the ADOS) and the person’s ‘internal’ status (dispositional traits measured by the AQ and social cognitive capability measured by the RMET).

We found that the operationalized camouflaging measure was not significantly correlated with age, VIQ, PIQ or FIQ. On average, women with autism showed more camouflaging than men with autism, but there was substantial variability in both groups. Greater camouflaging was associated with more depressive symptoms in men with autism and better signal-detection sensitivity in women with autism. The brain volumetric associations with camouflaging were largely sex/gender-dependent.

Following a positivist approach, our first aim was to quantitatively describe camouflaging in autism through an operationalization using existing measures. We acknowledge that our operationalization is not a direct measurement of camouflaging, which, in our view, could only be created in a valid way after careful concept formation via a qualitative (e.g. grounded theory) research approach (Glaser and Strauss, 2009). Nevertheless, our operationalization (i.e. the *external–internal discrepancy*) is a first step and may provide a reasonable proxy.

Using this proxy measure, which follows a normal distribution in our sample, we observed that individual differences in the degree of camouflaging were independent of age and IQ, in men and women with autism without intellectual disability. This suggests that the extent of camouflaging in adults with autism does not merely mirror degree of experience (reflected in chronological age). If camouflaging is partly learnt, one might expect a correlation with age/experience at younger ages. Recent studies have alluded to the presence of camouflaging in teenage (Head et al., 2014; Tierney et al., 2016) or even school age years (Hiller et al., 2014; Rynkiewicz et al., 2016). The developmental course of camouflaging remains to be revealed by longitudinal studies. In particular, how it affects one’s clinical experience (e.g. getting a timely autism diagnosis, developing mental health challenges) should be a focus of investigation. We also surprisingly found no significant association between camouflaging and VIQ, PIQ and FIQ in either men or women with autism. This suggests in this population, the extent of camouflaging does not merely reflect general reasoning ability or speed of processing. Instead, it may be more specifically associated with particular aspects of cognitive ability (discussed below) or personality, motivational or contextual factors.

We observed an on-average higher extent of camouflaging in women than men with autism of rather large effect size (Cohen’s *d* = 0.98). This fits well with previous findings from contrasting current ADOS and childhood ADI-R scores in men and women separately (Lai et al., 2011). It also corresponds well with the reports from women with autism and their parents as well as expert clinician’s observations (summarized in ‘Introduction’ section). Here, we are not able to delineate what contributes to this difference between men and women, but we suspect that socio-cultural factors, in particular gender-based expectations and gender socialization across development, may be key players (Kreiser and White, 2014). For example, protective same-gender friendship (i.e. being ‘mothered’) may conceal a girl or woman’s social difficulties; gender-based expectations may prompt a girl or woman with autism to ‘act like a girl/woman’ and ‘be more social’, and she may therefore develop higher censuring of own behaviours and more imitation or emulation of gender-normative social behaviours. Behavioural components contributing to the presentation and developmental course of camouflaging should be explored via qualitative research (Tierney et al., 2016) as an immediate future research direction (Lai et al., 2015).

It is important to note from our data that although camouflaging might have been portrayed as an integral part of the ‘female-phenotype of autism’, it is not specific to females. Even with sex/gender differences of large effect, the distributions of camouflaging score overlapped substantially between men and women with autism: there were women who showed little camouflaging and men who presented marked camouflaging (Figure 1(a)). On average, a sex/gender difference in camouflaging is evident, but it should be viewed as a phenomenon reflecting individual differences in social coping, rather than a diagnostic behavioural pattern distinguishing females versus males with autism at an individual level.

We tested the hypotheses that higher camouflaging is associated with higher anxiety and depression, and cognitively with better verbal ability, better signal detection from background events and more conservative responses. Findings confirmed some of the hypotheses, yet in a potentially sex/gender-dependent manner. When taking sex/gender into consideration, trend-level significant sex/gender-differential correlation patterns were observed between camouflaging and the clinical and cognitive correlates.

We predicted that camouflaging is exhausting and brings excessive stress and, therefore, may be associated with anxiety and depressive symptoms. Based on the background that men and women with autism in this study showed no differences in either symptom scores (but both had elevated scores approaching clinical range; Table 1), we found a pattern in support of the prediction for depressive symptoms in men (*r* = 0.533, *p* = 0.002) but not women with autism (*r* = 0.030, *p* = 0.876); we found no significant relationship between camouflaging and anxiety symptoms in either sex or gender. The nature of this study does not allow for testing causal relationships, yet based on the cross-sectional correlational patterns we suspect that the lack of association with anxiety might indicate that camouflaging in adults is an already adapted behavioural pattern. Investigation into the child and youth autistic population is necessary to address any potential associations between camouflaging and anxiety at younger ages. The positive association with depressive symptoms (as predicted) in men raises the possibility that they are more susceptible to the burden of camouflaging than women with autism are – perhaps women would have had more practice with, and might be better adapted to, implementing camouflaging due to gender-related social experience and demands. These ideas await rigorous investigation of the causal relationships between stress, anxiety/depression, cognitive features, camouflaging and social adaptation, using structural equation modelling (for cross-sectional data) or longitudinal designs.

The association between camouflaging and cognitive performance may shed light on potential cognitive underpinnings of camouflaging. Lehnhardt et al. (2016) suspected that verbal abilities might serve an important role for males with autism when it comes to camouflaging. Contrary to this prediction, we did not find a significant correlation between verbal ability and camouflaging in either men or women with autism. This suggests that the extent of camouflaging does not merely reflect verbal knowledge or reasoning; rather, it might be associated with verbal skills beyond these or might be underpinned by other cognitive capabilities.

We then examined performance on a response inhibition Go/No-Go task because among our available measures in this project, the SDT parameters from the Go/No-Go task most closely reflect the theoretical constructs of interest (i.e. one requires sensitive real-time monitoring of the environment, and/or a cautious, conservative response strategy, to successfully camouflage). The background is that men and women with autism in this study equally showed on-average poorer sensitivity in detecting signal from background compared to neurotypical controls, but were no more liberal or conservative in response strategy (see a previous study on a slightly larger but highly overlapping sample (Lai et al., 2012)). In this context, we again found a potentially sex/gender-differential pattern. Women (but not men) with higher camouflaging showed better signal-detection sensitivity, whereas there was no significant association between camouflaging and response strategy in either sex or gender. Lehnhardt et al. (2016) alluded to possible sex/gender-differential cognitive underpinnings of camouflaging by the indirect evidence that women with autism show higher processing speed and better executive functions (mainly in trail-making and verbal fluency tests) than men with autism. Our findings echo this suggested association between executive functions and camouflaging in women with autism by directly showing a predicted relationship. Again, causal inference cannot be made: it could be the case that better signal-detection supports and prompts more camouflaging or that more frequent camouflaging enhances cognitive control and signal-detection sensitivity. In sum, the converging message points to the need for studying the relationships between camouflaging and executive functions, particularly in females. Whether this is equally important in males with autism is unclear. Direct hypothesis testing concerning the cognitive bases of camouflaging (and the examination of sex/gender-differential relationships) is much needed.

Our last aim was purely exploratory and hypothesis generating. When testing for association between regional GM volume and camouflaging, we found statistically significant sex/gender-dependent association patterns while not finding any region that showed a significant overall correlation with camouflaging across sex/gender at the same statistical threshold. This indicates that the neuroanatomical association of camouflaging in autism may be largely sex/gender-dependent, particularly around the medial temporal and cerebellar structures. When dissecting the sex/gender-differential pattern, we found a lack of association in males but a significant negative correlation in females (i.e. the higher camouflaging, the smaller regional volume).

When using the Neurosynth Image Decoder for reverse inference (Gorgolewski et al., 2016; Poldrack, 2006), that is, to identify scientific terms in the ‘big data’ of the neuroscience literature mostly associated with the voxels showing significant correlation with camouflaging in women with autism, we found anatomical terms (e.g. cerebellum, medial temporal lobe, para/hippocampus, amygdala) as well as cognitive terms about emotion and memory. This exploratory, hypothesis-generating approach, in association with the cognitive findings regarding executive functions (for which the cerebellum is closely involved), gives candidate neurocognitive components for future hypothesis testing to uncover the bases of camouflaging, particularly for females.

As the first study operationalizing and quantifying camouflaging, the findings should be considered exploratory and have to be interpreted with caution, keeping in mind the following limitations. First, camouflaging was quantified by a mathematical manipulation of available measures based on our operationalization as *the discrepancy between the person’s ‘external’ behavioural presentation in social–interpersonal contexts and the person’s ‘internal’ status*. Factors potentially affecting scoring of these contributing measures will have impacts on the derived camouflaging measure, and therefore, findings need to be interpreted considering these potentially confounding factors. For example, social communication behaviours measured by the ADOS may be affected by one’s anxiety level during the assessment, and gender stereotype of the examiner may affect how behaviours are scored; dispositional traits measured by the AQ may be influenced by one’s self-referential ability and even intuitive/automatic masking of difficulties; social cognitive ability measured by the RMET may be affected by one’s lexicon and verbal abilities. Additionally, although the content validity of the camouflaging measure is ensured based on the concept of *external–internal discrepancy*, whether this measure shows satisfactory concurrent validity awaits comparisons with future studies that also quantify camouflaging. For example, when a substantially large sample is available, quantifying camouflaging by regression methods (e.g. the residuals after regressing out the variances of ‘internal/actual’ characteristics from ‘external’ behavioural manifestations) could provide another metric to be examined. In addition, instead of operationalizing camouflaging as external–internal discrepancy, one could also operationalize it by social imitation capacities based on the hypothesis that camouflaging heavily involves social imitation and adaptation; these capacities could be measured by components of well-established instruments such as the Self-Monitoring Scale (Snyder, 1974), its revision (Lennox and Wolfe, 1984) and the Multidimensional Iowa Suggestibility Scale (Kotov et al., 2004).

Second, all analyses of the relationships between camouflaging and clinical symptoms, cognitive abilities and regional brain volume were correlational in nature, and no causal relationships could be inferred. Mechanisms discussed above are speculations and have to be tested using longitudinal or intervention designs or hypothesis-based modelling with more comprehensive data collection relevant to this topic.

Third, due to the limitation of the dataset (i.e. we did not perform ADOS for the control sample in the cohort), we were not able to compare sex/gender difference in camouflaging in autism in the context of probable neurotypical sex/gender difference. If there is an underlying neurotypical sex/gender difference, then the findings need to be interpreted accordingly (Lai et al., 2015). This further examination is crucial but will be available only with studies capable of quantifying camouflaging in neurotypical individuals, based on the same operationalization (i.e. the external–internal discrepancy) but using measures other than the ADOS that can quantify social communicative behaviour and are sensitive enough to pick up individual differences in the neurotypical population.

Fourth, the findings are derived from a moderate-sized adult sample with autism and without intellectual disability. The extent to which the findings generalize to the full autism spectrum has to be further examined. We suspect that beyond individual general and specific cognitive factors, one’s personality, social experience and developmental stage (which are associated with age-relevant social demands), as well as the socio-cultural context, will all have particular influences.

In conclusion, this study provides a first attempt to operationalize and quantify camouflaging in men and women with autism, showing substantial inter-individual variability but on-average higher levels in women than men, and demonstrates potentially sex/gender-dependent associations with depressive symptoms, signal-detection sensitivity and regional brain volume. We urge more investigations into this clinically important phenomenon to better delineate the construct. Ideally, this should include (1) qualitative (or mixed-design) approaches to reveal first-person account and second/third-person observation about what triggers (e.g. when and why one camouflages) and constitutes camouflaging (e.g. what the behavioural components are, and which of them are automatic/intuitive vs requiring one to act/perform with effort, and which of them are simply masking vs compensating); (2) psychological studies to understand the personality, cognitive and contextual bases of camouflaging; and (3) clinical studies to assess the positive and negative consequences of camouflaging, as well as how camouflaging has an impact on the diagnosis of autism (e.g. whether higher levels of camouflaging result in delayed or missed diagnosis) and the identification of relevant clinical issues. These studies will benefit from examining camouflaging-related factors not only in individuals currently having a clinical diagnosis of autism but also those who may be on the spectrum (e.g. those having high-level autistic-like traits and/or social adaptation difficulties yet who have failed or have not yet to be diagnosed with autism), in order to inform how camouflaging may have a real-world healthcare impact. A thorough understanding of camouflaging in autism may improve the diagnosis of autism across sex/gender, the identification of needs and assets for each person and the tailored individualized supports.
